# P02.74. Treating Generalized Anxiety Disorder (GAD) using a self-care model of Complementary and Alternative Medicine (CAM) therapy

**DOI:** 10.1186/1472-6882-12-S1-P130

**Published:** 2012-06-12

**Authors:** F McPherson, L McGraw

**Affiliations:** 1 Internal Medicine Clinic, Madigan Army Medical Center, JBLM, Lacey, USA

## Purpose

To investigate the effectiveness of a pilot program using a CAM multi-therapy treatment program focusing on self-care behaviors for the treatment of Generalized Anxiety Disorder (GAD).

## Methods

This is a quasi-experimental one-group pretest-posttest design using a convenience sample of volunteers at a military treatment facility in the Pacific Northwest. Participants (N=37) were enrolled if they had a documented history of GAD or met screening criteria for GAD using the GAD-7, with 68% completing the program (N=25). Participants received acupuncture treatments one time/week for six weeks and were asked to engage in yogic breathing exercises, self and/or partner assisted massage therapy using scented oils, episodic journaling, nutrition counseling, and exercise.

## Results

Significant reductions were identified on pre and post GAD-7, Depression-Anxiety-Stress Scale-21, UCLA Loneliness Scale, and a significant increase was noted in the Rosenberg Self-esteem Scale. In addition patient behavior (participation in study therapies) remained consistently high and a secondary outcome was reduction in anti-anxiety medication use.

## Conclusion

The findings in this pilot study suggest multimodal interventions to facilitate self-care is feasible and that a multi-therapy treatment program using CAM therapy, focusing on self-care behaviors may be an effective adjunct therapy for the treatment of Generalized Anxiety Disorder.

